# Salivary mycobial profiles of oral cancer patients – a cohort study

**DOI:** 10.1080/20002297.2026.2696598

**Published:** 2026-07-30

**Authors:** Anna I. Mäkinen, Derek W. K. van der Plas, Mark J. Buijs, Antti A. Mäkitie, Jukka H. Meurman, Egija Zaura, Bernd W. Brandt

**Affiliations:** a Department of Oral and Maxillofacial Diseases, University of Helsinki and Helsinki University Hospital, Helsinki, Finland; b Department of Preventive Dentistry, Academic Centre for Dentistry Amsterdam (ACTA), Vrije Universiteit Amsterdam, University of Amsterdam, Amsterdam, the Netherlands; c Department of Otorhinolaryngology – Head and Neck Surgery, University of Helsinki and Helsinki University Hospital, Helsinki, Finland; d Research Program in Systems Oncology, Faculty of Medicine, University of Helsinki, Helsinki, Finland; e Division of Ear, Nose and Throat Diseases, Department of Clinical Sciences, Intervention and Technology, and Karolinska Institutet, Karolinska University Hospital, Stockholm, Sweden

**Keywords:** Squamous cell carcinoma of head and neck, *Candida*, *Malassezia*, mycobiome, mycobiota, follow-up studies, mycotype

## Abstract

**Background:**

Several studies have investigated the possible links between oral yeast infections and oral cancer. However, many of these studies concentrate on single species or genera and the overall oral mycobial profiles of oral cancer patients are not well known.

**Objective:**

We examined the salivary mycobial profiles of oral squamous cell carcinoma (OSCC) patients before and after cancer treatment and compared them with those of non-cancer controls. Additionally, we tested the recently introduced concept of two salivary mycotypes—one driven by the genus *Candida* and one by the genus *Malassezia*—in the context of OSCC.

**Design:**

Paraffin-stimulated saliva from OSCC patients before cancer treatment (*n *= 75), after a mean follow-up of 4 years (*n *= 27) and from non-cancer controls (*n *= 81) was analysed using ITS2 amplicon sequencing.

**Results:**

The OSCC patients had lower alpha-diversity (*p* = 0.001, Shannon diversity index) and higher relative abundance of OTU1 *Candida albicans* compared with controls. No significant difference in beta-diversity or in differential abundance of OTUs was found between the paired pre-treatment OSCC and follow-up samples or the follow-up samples and controls. Baseline OSCC patients had higher prevalence of the *Candida-*mycotype compared with controls (*p* = 0.019). The prevalence of the *Malassezia*-mycotype increased from baseline OSCC to follow-up.

**Conclusions:**

Our results suggest that OSCC patients have a *Candida*-dominated salivary mycobiota that differs significantly from that of non-cancer controls and shifts towards an increased prevalence of *Malazzesia* after cancer is treated.

## Introduction

The role of the oral microbiome in the development and progression of head and neck cancers is increasingly recognized, as different bacterial, viral and fungal species have been associated with the presence or absence of cancer. In the case of oral cavity squamous cell carcinoma (OSCC) specifically, studies on the bacterial ecology of tumours have been gaining momentum. However, only a handful of studies on the mycobiota of these patients exist to date.

The link between oral *Candida* infections and oral cancer was suggested by Cawson and Lehner in the mid-1960s [1]. Since then, multiple studies have found a high prevalence of *Candida* in patients with OSCC [2–4]. Several studies have suggested different pathways in which these yeasts may contribute to OSCC risk—from their ability to produce carcinogenic amounts of acetaldehyde [5,6] to *Candida albicans'* capability to facilitate mucosal penetration of certain bacteria linked to cancer development, such as *Porphyromonas gingivalis* [7]. The spectrum of fungal species identified from the oral cavity has increased since high-throughput sequencing methods have become mainstream, and a so-called core oral mycobiota consisting of 13 to 14 genera has been suggested by two separate groups [8,9]. Additionally, the concept of two ecologically distinct mycotypes, one driven by *Candida* species and the other driven by the genus *Malassezia*, was recently introduced for the salivary mycobiota [10]. Specifically, the *Candida*-driven mycotype has been positively associated with non-oral cancer, smoking and steroid use as well as caries [10]. While previous studies have largely been dedicated to investigating single species or single genera, exploring the mycobiota in a broader range may give us new openings regarding the associations between species, genera and OSCC.

In this mixed cohort and case‒control study, our objective was to study the possible differences in the salivary mycobiota of OSCC patients and non-cancer controls. To achieve this, we collected saliva samples from OSCC patients before cancer treatment and compared their salivary mycobial profiles with those of non-cancer controls. We also compared the salivary mycobial profiles of these patients before treatment and after post-treatment follow-up time to study whether treating oral cancer affects the salivary mycobial profile in the long term. Furthermore, we tested the concept of *Candida-* and *Malassezia*-driven mycotypes in the context of OSCC patients. We hypothesized that the mycobial profiles differ between all these study groups based on the assumption that both having oral cancer and treating it affect oral ecology.

## Patients and methods

### Sample collection

#### Study subjects

This study is part of a larger study on the mycobial profiles of OSCC patients before and after cancer treatment. The original study group consisted of 100 OSCC patients referred for cancer treatment with the official national treatment protocol at the Helsinki University Hospital in Helsinki, Finland, between 2011 and 2014. The inclusion criteria were cancer of squamous cell origin within the oral cavity with upcoming surgical removal of the tumour. The exclusion criteria were cancer of the lip, tonsil, larynx and/or pharynx; tumours of non-squamous cell origin, and the patient's inability to give informed consent. The patients gave their sample before receiving any cancer treatment, i.e. the surgical removal of the tumour and administration of any neoadjuvant or adjuvant therapy. Forty-four of these patients continued the study after an average follow-up time of 4 years, at which point a follow-up sample was collected. The reasons for discontinuation in the study included moving to another hospital district's catchment area (*n* = 8), unwillingness to continue participation (*n* = 11), or death (*n* = 37).

Additionally, 103 controls were recruited from the Helsinki University Hospital Department of Otorhinolaryngology—Head and Neck Surgery outpatient clinic and the Helsinki Day Activities Centre for the Elderly. The inclusion criterion for the controls was an age between 35 and 100 years, with the aim of creating an age- and sex-matched control group for the case‒control study, and the exclusion criteria were previously treated or currently diagnosed or suspected head and neck cancer. Owing to the strict processing and quality requirements for the sequencing data, we were unable to strictly match the number of cases and controls but rather opted for age- and sex-matching of the groups.

The basic characteristics of the patients, including age, sex, smoking status, whether they use alcohol edentulism, as well as tumour and treatment related data were collected from hospital records both before cancer treatment and at follow-up. Matching basic characteristics of the controls were collected via a structured questionnaire.

#### Saliva samples

Paraffin-stimulated whole saliva samples were collected from all patients and controls according to Mäkinen et al. [11], and the stimulated salivary flow rate (SFR) was recorded as ml/min. All samples were stored on ice immediately after collection and delivered to the Department of Oral and Maxillofacial Diseases Laboratory at the University of Helsinki, Helsinki, Finland, for storage. After cultivation of the samples on CHROMagar™ Candida (CHROMagar™, Paris, France) to establish *Candida* growth (CFU/ml) as described in Mäkinen et al. [4], up to 2 ml of each sample was stored at −80 °C before delivery in dry ice to the Academic Centre for Dentistry Amsterdam (ACTA), Amsterdam, the Netherlands, for mycobiota analyses. In total, 236 samples had enough saliva for sequencing after cultivation (0.3 ml or more).

### Sample processing

#### DNA isolation

DNA isolation was done in batches of 84 samples. To control for potential contaminations of kit chemicals, isolation blanks, i.e. 200 µl of DNA-free water, were added to each batch. The saliva samples were thawed and vortexed extensively, and 200 μl was transferred to an assigned well in a 1.1-ml deep-well plate containing 250-μl 0.1-mm Zirconia beads (BioSpec Products, Inc. Bartlesville, OK, USA), 200 μl of phenol (Rotiphenol, Carl Roth GmbH & Co. KG, Germany) and 100 μl of lysis buffer (MagMini DNA isolation kit, LGC Genomics Ltd., Hoddesdon, UK). After sealing, the deep-well plate was placed in a MiniBeadBeater-96 (BioSpec Products, Bartlesville, OK, USA) and subjected to four times 2 min bead beating at 2100 oscillations/min. DNA extraction and purification were done using the MagMini DNA Isolation Kit (LGC Genomics Ltd., Hoddlestone, UK). To quantify the bacterial DNA yield, the DNA was subjected to a qPCR with universal primers specific to the bacterial 16S rRNA gene [12]. For fungal DNA yield, primers and probes specific to the fungal 18S rRNA gene were used [13].

#### Fungal amplicon preparation and sequencing

Fungal amplicons of the Internal Transcribed Spacer region 2 (ITS2) were generated through PCR with the following primers: gITS7ngs forward: GTGARTCATCRARTYTTTG, and ITS4ngs reverse: TCCTSCGCTTATTGATATGC [14]. The fungal primers contained adapter sequences to ligate to Nextera Set A and Set B Amplicon primers with 8-nucleotide index sequences (Illumina protocol, Part # 15044223 Rev. B). The PCR products were purified using AMPure XP beads (Beckman Coulter). The Nextera adapters were ligated using an 8-cycle PCR (Illumina protocol, Part #15044223 Rev. B). After purification with AMPure XP beads, the PCR yield of each sample was determined with the Quant-iT PicoGreen dsDNA Assay Kit. Next, an equimolar pool containing all samples was made, and the quality and size of the amplicon were determined on an Agilent TapeStation with an Agilent ScreenTape D1000 (Agilent Technologies, Santa Clara, CA, USA).

Paired-end sequencing (2 × 301 nt) of the ITS2 region was conducted on the MiSeq platform (Illumina, San Diego, CA, USA) using the MiSeq Reagent Kit v3 at the Amsterdam University Medical Center, the Netherlands. The flow cell was loaded with 8 pmol of DNA containing 30% PhiX.

#### Processing of sequencing data

The sequence data were processed as described in Vidal et al. [15]. Briefly, paired-end reads were merged and quality-filtered using USEARCH (version 8.0.1623 [16]) and clustered into 97% operational taxonomic units (OTUs), after which the sequences were mapped to the OTU centroids. The most abundant sequence of each OTU was selected and assigned a taxonomy using the RDP classifier [17] with UNITE version 9.0 (Fungi, sh_refs_qiime_ver9_dynamic_s_25.07.2023.fasta, after extracting the ITS2 region) as a taxonomic reference [18]. To study for possible contamination within the samples, the decontam R-package (version 1.20) was used [19]. Based on these results, two OTUs (OTU2 and OTU417, both assigned the genus *Purpureocillium*), with extremely low *p*-values (1.4E-15 and 3.3E-11, respectively), were removed. Next, all samples with less than 1000 reads were discarded (*n* = 30 samples removed). Additionally, samples with a value for 18S rRNA gene qPCR concentration was lower than that of the highest isolation control were removed because they were considered to have a too low fungal DNA yield (*n* = 23 samples removed). Furthermore, only samples that had respective patient samples after processing were included for longitudinal analyses of pre-treatment and follow-up samples (20 patients, *n* = 40 samples).

### Statistical analyses

The alpha diversity indices Shannon, Chao1, species richness and Simpson were calculated as the median values per index over 100 random subsamples (without replacement) of 1000 reads per sample (estimate_richness–function, phyloseq (v1.44), R version 4.3.1, [20,21]. The OTU data was then transformed by centred log-ratio transformation (CLR) for each group comparison using the microbiome R package (v1.22 [22]) after removing OTUs with zero reads. Principal component analysis (PCA, Euclidean distance) and permutational analysis of variance (PERMANOVA; 99999 permutations; Euclidean distance) of the cross-sectional and longitudinal data were calculated using the CLR-transformed data in PAST software version 4.12 [23]. Differential abundance analyses were done using the linear discrimination analysis (LDA) effect size (LEfSe) [24] on data subsampled at 1000 reads per sample, as well as on non-subsampled data using ALDEx2 (version 1.32.0 [25,26]).

Differences between groups were analysed using IBM SPSS Statistics version 28, with the continuous variables SFR, salivary *Candida* concentration (log10[CFU/ml]), fungal 18S rRNA gene concentration (ng/µl) and bacterial 16S rRNA gene concentration (ng/µl), as well as the alpha diversity indices Shannon, Simpson, Chao1 and species richness tested after determining the normality of the data (independent samples *t*-test or independent samples Mann‒Whitney U test for cross-sectional data; related-samples *t*-test or paired-samples Wilcoxon signed rank test for longitudinal data). Chi-square test was used for the categorical confounding variables sex, smoking, alcohol use and edentulism, and the independent samples Mann‒Whitney U test was used for the continuous confounding variable age.

The samples were assigned either the *Candida* mycotype or *Malassezia* mycotype by calculating the relative abundance of the genus *Candida* and the genus *Malassezia* in each sample and determining which genus had a higher relative abundance. OTUs assigned to either the genus *Candida* or the genus *Malassezia*, as well as OTUs which have previously been known as clinically relevant *Candida*, were used in generating the mycotypes (Supplementary information Table 2). Samples that had a difference in relative abundance at the genus level of less than 5% between the genera assigned to the two mycotypes were removed from the mycotype analyses due to ambiguity in the assignment of the mycotype.

## Results

**Figure 1.**
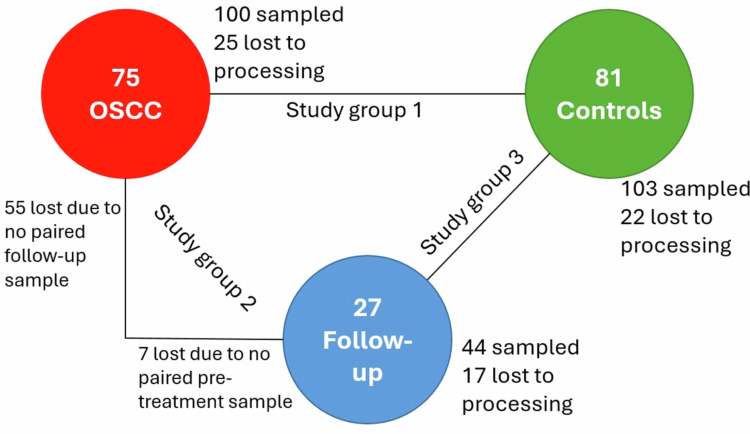
Visualization of the study groups. The paired pre-treatment samples in study 2 are included in the group of baseline OSCC samples (*N* = 75) in study 1, and the paired follow-up samples in study 2 are also included in the 27 follow-up samples in study 3.

Altogether, 236 samples were sequenced, and 183 of these, 183 were available for statistical analyses after sequence data processing. Among the 183 samples, 75 were pre-treatment samples from OSCC patients, and 81 were from non-cancer controls available for a case‒control study. Additionally, a total of 27 follow-up samples were available for analyses after processing; however, after pairing the follow-up samples with the pre-treatment samples data, only 20 patients had data from both pre-treatment and follow-up timepoints. The groups investigated are shown in Figure 1, and the basic characteristics of the study subjects are given in Table 1.

**Table 1.** Basic characteristics of the different groups studied. Difference in mean age was tested using independent samples Mann–Whitney U for both OSCC and follow-up (*N* = 27) against control, while paired sample *t*-test was used to test the difference between paired pre-treatment and follow-up (*N* = 20) samples. Staging and grading of tumours according to American Joint Committee on Cancer (AJCC) Cancer Staging Manual 7th edition, 2010.

		OSCC	Control	*p*	Paired pre-treatment	Follow-up	*p*	Follow-up	*p^*
		(*N* = 75)	(*N* = 81)		(*N* = 20)	(*N* = 20)		(*N* = 27)	
Mean age, years		68.7	67	ns	65.5	69.7	ns	69.8	
Female sex		34 (45%)	44 (54%)	ns	8 (40%)		ns*	13 (48%)	ns
Edentate		10 (13%)	4 (5%)	ns	0 (0%)	2 (10%)	ns	4 (15%)	ns
Smoker		35 (47%)	8 (10%)	<0.001	8 (40%)		ns*		
Alcohol user		48 (64%)	47 (58%)	ns	16 (80%)		ns*		
Size of primary tumour									
T1		33 (44%)			13 (65%)		ns*		
T2		19 (25%)			3 (15%)		ns*		
T3		3 (4%)			1 (5%)		ns*		
T4		20 (27%)			3 (15%)		ns*		
Stage of cancer									
I		27 (36%)			11 (55%)		ns*		
II		15 (20%)			2 (10%)		ns*		
III		1 (1%)			1 (5%)		ns*		
IV		32 (43%)			6 (30%)		ns*		
Grade^#^									
1		16 (21%)			4 (20%)		ns*		
2		47 (63%)			14 (70%)		ns*		
3		10 (13%)			2 (10%)		ns*		
Location of cancer (ICD-10)^¤^									
C02		23 (31%)			7 (35%)				
C03		24 (32%)			3 (15%)				
C04		17 (23%)			5 (25%)				
C05		3 (4%)			2 (10%)				
C06		8 (11%)			3 (15%)				

^*^ Paired pre-treatment samples tested against the original set of OSCC patients.

^^^ Follow-up samples (*N *= 27) tested against the controls.

^#^ Two samples had a tumour of an unknown grade.

^¤^ ICD-10 = International Classification of Disease 10th revision version 2016; C02= other and unspecified parts of the tongue (anterior 2/3 of the tongue); CO3 = gingiva; C04 = floor of mouth; C05 = palate; C06 = other and unspecified parts of the oral cavity (including cheek mucosa, vestibules of mouth and retromolar area).

### Study group 1: patients (*n* = 75) and controls (*n* = 81)

**Figure 2.**
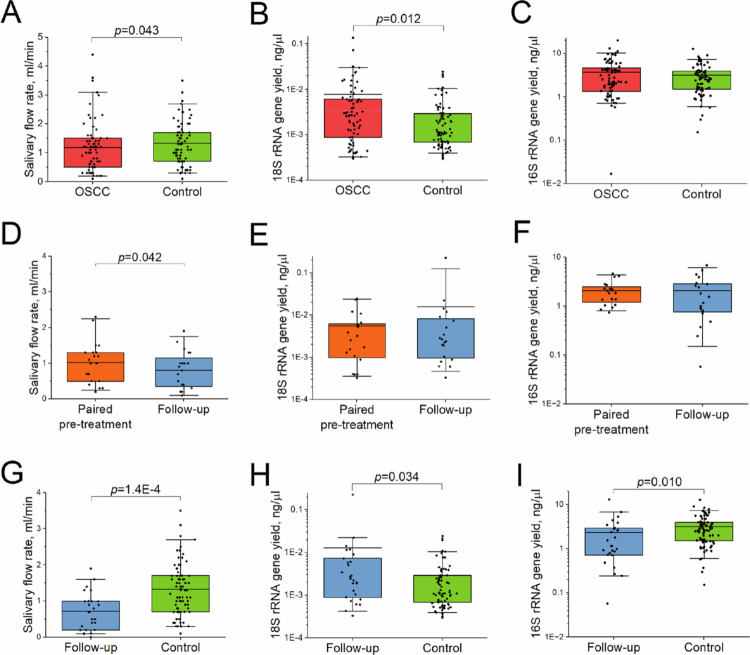
Salivary flow rate (A, D, G), fungal DNA yield (B, E, H) and bacterial DNA yield measures (C, F, I) of OSCC compared to controls (A–C), paired pre-treatment and follow-up samples compared together (D–F) and follow-up samples compared to controls (G–I). The cross-sectional comparisons of OSCC (A–C) and follow-up samples (G–I) against controls were done using independent samples Mann–Whitney U, and the longitudinal comparison between paired pre-treatment and follow-up samples was done using paired samples *t*-test (D) and the Wilcoxon signed rank test (E, F).

The OSCC group had significantly more smokers compared with the controls (Table 1). Additionally, the OSCC patients had a significantly lower mean SFR and higher mean fungal DNA yield (Figure 2A and B). The groups did not differ significantly with regards to alcohol drinking, being edentate or mean bacterial DNA yield.

**Figure 3.**
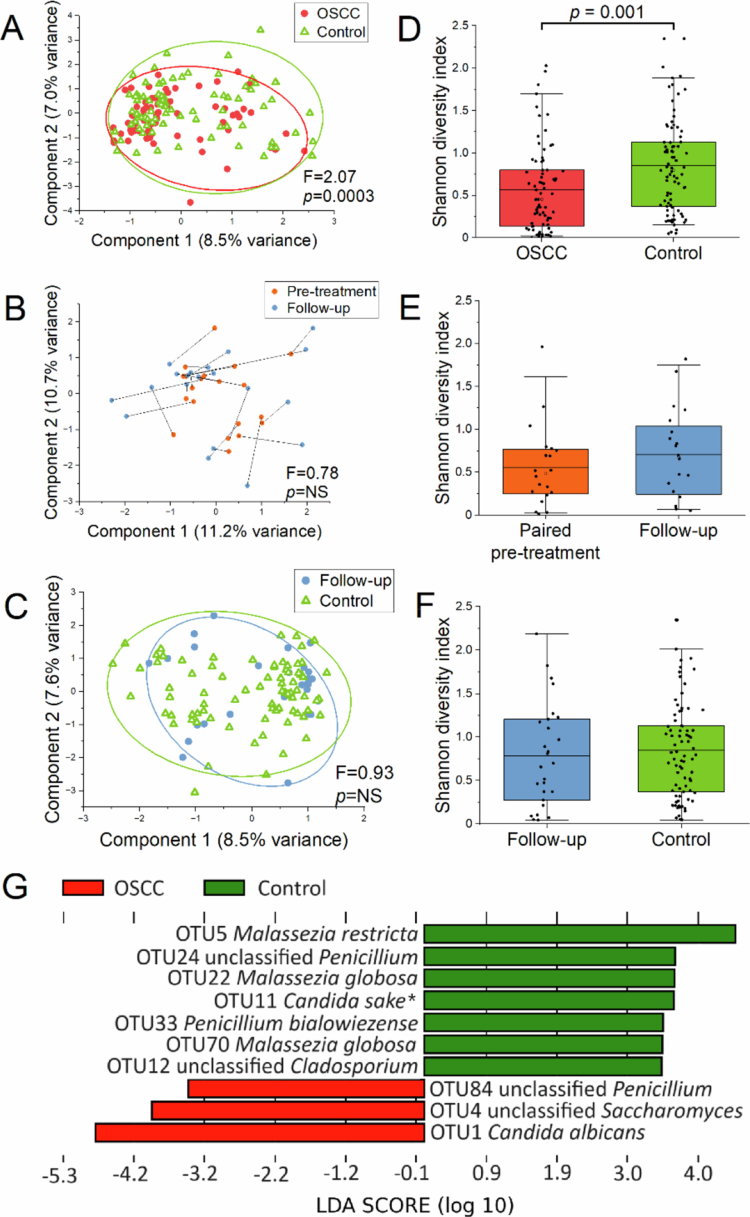
Differences in the mycobial profiles of OSCC patients compared to controls and after follow-up. (A) Principal component analysis (PCA) plot of the pre-treatment samples compared to the controls, F- and *p*-values from PERMANOVA. (B) PCA plot of the paired pre-treatment samples compared to the follow-up (*n* = 20) samples (F- and *p*-values from repeated measures PERMANOVA). (C) PCA plot of the follow-up samples (*n* = 27) compared to the controls (F- and *p*-values from PERMANOVA). (D–F) Median Shannon diversity values over 100 subsamples per sample; the difference is significant between OSCC patients and controls (Independent-samples Mann‒Whitney U test, D), but not between pre-treatment and follow-up samples (Related-samples Wilcoxon Signed Rank test, E) or follow-up and controls (independent-samples Mann–Whitney U, F). (G) Differential abundance analysis comparing OSCC and controls (LEfSe). *Taxonomy reassigned to *C. sake* from *Debramycetaceae* sp. (UNITE v9 database) due to identical sequence as compared with NCBI BLAST and UNITE database.

The mycobial profiles of the OSCC patients differed statistically significantly from those of the controls (Figure 3A). The Shannon and Simpson alpha-diversity indices were statistically significantly lower among the OSCC patients (*p* = 0.001 for both independent samples, Mann‒Whitney U, Figure 3D, data on Simpson's diversity index not shown), while no statistical significance was found using Chao1 or species richness indices.

When differential abundance analysis was performed using LEfSe, a significantly higher abundance of OTU1 *C. albicans* was found among the OSCC patients, whereas OTU5 *Malassezia restricta* and OTU22 *Malassezia globosa* were significantly more abundant among the controls (Figure 3G). However, a more robust differential abundance analysis with ALDEx2, after multiple testing correction, found no significant OTUs that differed between the groups.

Additionally, a subpopulation of non-smoker OSCC patients (*n* = 40) and controls (*n* = 70) was studied as a consideration for the significantly different number of smokers in the patient and control groups. Again, the mycobial profiles were statistically significantly different (F = 2.01, *p* = 8.2E-4, PERMANOVA, PC1 and PC2 explaining 16.3% of the variance, Supplementary information Figure 1A), and the Shannon and Simpson alpha-diversity indices among non-smokers were statistically significantly lower among OSCC patients compared with controls (*p* = 0.005 and *p* = 0.008, respectively, independent samples Mann–Whitney U, Supplementary information Figure 1B and C). Similarly to the whole group studied, the mean SFR was significantly lower among the OSCC compared with the controls (*p* = 0.021, independent samples Mann‒Whitney U, Supplementary information Figure 1D), but the difference in fungal DNA yield was not significant (Supplementary information Figure 1E).

### Study group 2: patients before and after treatment (*n* = 20)

The median follow-up time of the patients was 45.5 months (min 36 months, max 78 months). All patients included in this follow-up study were clinically cancer-free at the time of the second sampling. The subset of 20 patients included in the longitudinal study did not differ statistically significantly from the original set of 75 OSCC patients with regards to age, sex, smoking, alcohol drinking or being edentate.

The patients had a significantly lower SFR at follow-up compared to pre-treatment, but the fungal or bacterial DNA concentrations were not statistically significantly different (Figure 2D–F). The mycobial beta-diversity did not differ significantly between the two collection timepoints, either (Figure 3B). The fungal Shannon and Simpson alpha-diversity values were increased at follow-up compared with the pre-treatment samples (Figure 3E, data on Simpson's diversity index not shown), but the difference was not statistically significant. No difference was found when using alpha diversity indices Chao1 and species richness. Furthermore, no OTUs were found to be significantly different in relative abundance between the groups with either of the differential abundance analysis methods used.

### Study group 3: comparing follow-up samples (*n* = 27) to controls (*n* = 81)

The follow-up group had a significantly lower mean SFR than the controls (Figure 2G). Additionally, the follow-up group had a significantly lower bacterial DNA yield and a higher fungal yield than the controls (Figure 2H and I).

There was no significant compositional difference between the follow-up samples and the controls. Neither the alpha- nor the beta-diversities differed significantly between the follow-up samples and the controls (Figure 3C and F). Again, no OTUs were significantly different in their abundance between the groups when analysed using either of the differential abundance analysis methods.

### Differences between mycotypes

**Figure 4.**
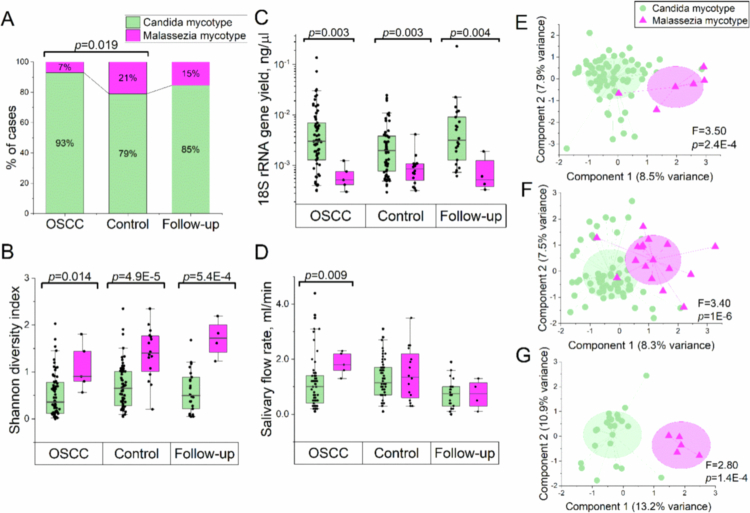
Differences between two different mycotypes within the three groups. (A) Prevalence of the two mycotypes within the groups. The difference in prevalence was significant between OSCC and controls (Fisher's Exact test). (B) Shannon diversity of the samples belonging to the different mycotypes. Within all the groups, the samples belonging to the *Malassezia* mycotype had a significantly higher alpha-diversity compared to the *Candida* mycotype (independent samples Mann–Whitney U). (C) Comparison of the fungal DNA yield between the two mycotypes within the three groups (independent samples Mann‒Whitney U test). (D) Comparison of the salivary flow rates (SFR) of OSCC patients, controls and patients at follow-up between the two mycotypes. The patients with the *Malassezia* mycotype had a statistically significantly higher SFR only in group OSCC (independent samples Mann‒Whitney U test). (E–G) Principal component analysis (PCA) plots comparing the two mycotypes within groups OSCC (E), controls (F) and follow-up (G).

Owing to ambiguity in the assignment of a mycotype in some of the samples, 176 samples were available for analyses of the differences in mycotypes, 72 of which were pre-treatment OSCC, 78 were controls, and 26 were follow-up samples. In all the groups, the *Candida* mycotype was more prevalent than the *Malassezia* mycotype (Figure 4A), but the control group did have statistically significantly more *Malassezia* mycotype compared to the OSCC group (*p* = 0.019, Fisher's Exact test). The prevalence of the *Malassezia* mycotype was, however, not statistically significantly different when comparing the follow-up group to either the pre-treatment OSCC samples or the controls.

Within all the groups studied, the mycobial beta-diversity differed significantly between the two mycotypes (Figure 4E–G). In addition, the Shannon diversity index was significantly higher in samples with a *Malassezia* mycotype within all groups (Figure 4B). Furthermore, the fungal DNA yield was significantly lower in the *Malassezia* mycotype compared to the *Candida* mycotype (Figure 4C). Within the OSCC group, patients with a *Malassezia* mycotype had a significantly higher SFR (Figure 4D), but no significant difference was found within the follow-up group or the controls. Furthermore, smoking, alcohol drinking or being edentate were not statistically significantly associated specifically with either of the mycotypes in any of the groups (Supplementary information Table 1).

## Discussion

We studied the salivary mycobiota of patients with OSCC and compared the findings cross-sectionally to non-cancer controls and longitudinally to a subgroup of these patients after their cancer treatment. We hypothesized that the mycobial profile would be different from that of controls both due to cancer and treating it. We also studied the concept of two mycotypes in the context of oral cancer.

The results showed that, before cancer treatment, OSCC patients had significantly more fungal DNA in their saliva and a lower fungal diversity than the non-cancer controls, which might explain the significant difference in fungal composition. The difference in the mycobial profiles was evident even when smokers were removed from the pre-treatment and control datasets. However, in the non-smoker dataset, there was no statistically significant difference in the salivary fungal DNA yield between the OSCC and control groups. Thus, the difference in fungal DNA yield is likely due to the effect of smoking rather than the OSCC environment of the patients. The differential abundance analysis showed a significantly higher abundance of *C. albicans* among the OSCC patients compared to the controls; however, with more robust statistical analysis using multiple testing correction, the significance was lost. Previously, *C. albicans* has been associated with oral cancer [27]. Additionally, a study of the ITS1 region found a significantly higher abundance of the genera *Clitopilus* and *Candida* in the saliva of OSCC patients compared to healthy controls, while genus *Morchella* was found to be the most abundant among healthy controls [28]. Neither the genera *Clitopilus* nor *Morchella* were present in our data.

The mycobial composition of the matched pre-treatment OSCC samples and follow-up samples did not differ significantly. The lack of statistical significance may be due to the low number of samples, since only 20 patients had samples from both pre-treatment and follow-up timepoints available for analyses. Interestingly, an increase in fungal alpha-diversity was seen at follow-up. Previously, higher fungal diversity has been reported in cases of disturbed oral or systemic health, such as in dentally compromised patients compared with those with better oral hygiene or patients with an eating disorder, compared with healthy controls [29,30].

The follow-up samples were also compared with the non-cancer controls to see whether the salivary mycobial profile of OSCC patients becomes more similar to the non-cancer profile after treatment. Indeed, there was no statistically significant difference in the mycobial alpha- or beta-diversities between these groups, nor were there any significantly differentially abundant OTUs that distinguished the groups from one another. The lack of statistical significance may, however, be due to the small number of follow-up samples compared to that of the controls rather than the salivary mycobial profiles becoming more like that of the controls. A larger group of treated OSCC patients is needed to verify these results.

The existence of two distinct mycotypes in saliva has recently been reported by at least two research groups [10,31]. When we studied the different mycotypes, we found that while the *Candida* mycotype was statistically significantly more prevalent among the OSCC patients compared with controls, there was no significant difference when compared the follow-up samples with either the paired pre-treatment samples or the controls. The *Malassezia* mycotype was found to be more diverse than the *Candida* mycotype in all groups, which was also the case in the study by Hong et al. (2020). In this study, we found that the samples with a *Malassezia* mycotype had a significantly lower fungal DNA yield than the samples with a *Candida* mycotype; in fact, among the pre-treatment OSCC patients, the fungal 18S rRNA gene concentration of all the samples categorized into the *Malassezia* mycotype was less than 1 pg/µl. Contrary to the study by Hong et al. (2020), where a statistically significant positive correlation between the *Candida* mycotype and smoking status was found, no association between smoking and the mycotypes was found in this study. It should be noted that the cohort in Hong et al. (2020) consisted of both healthy controls and non-oral solid tumour cancer patients undergoing chemotherapy, which may affect the reproducibility of their findings in this study.

When processing our data, we found that the mycobial data concentrated around only a few OTUs, with over 99% of the OTUs having fewer than 100 reads per sample. Furthermore, the fungal DNA yield in the samples was very low, with an average fungal 18S rRNA gene concentration ranging from 2.9 pg/µl among the controls to 13 pg/µl among the 27 cancer follow-up samples. Owing to the low fungal biomass in saliva in general, a proper quantification of the fungal DNA yield should always be included in salivary mycobiome studies and used as part of data filtering [32]. When assessing the validity of the data in this study, the abundance of potential contaminant OTUs became an issue, especially in samples which had a fungal DNA concentration of 1 pg/µl or less. Therefore, we suggest that the presence of contaminants should be carefully considered when drawing conclusions from sequenced samples that have a very low fungal biomass.

The strength of this study is the relatively large number of samples available for the analyses after setting strict criteria for data quality. Fifty-three samples were removed due to low quality reads, which may have affected the statistical power of the study but helped to ensure that all the results and their interpretation were done on high quality data. The number of follow-up samples included in this study was rather high, considering the low 5-year survival rate of around 50% among oral cavity cancer patients in general [33]. Furthermore, owing to the long follow-up time, most of the patients had been in recovery for several years before the second sampling, thus giving us the opportunity to study these patients both with and without cancer, even though any causational links are impossible to speculate. Our study population consists of a homogenous group of Caucasian ethnicity with similar age and sex distribution across all three groups. The limitation of this study is the lack of full dental records complete with periodontal status and information on dietary habits, which may affect the salivary mycobiome. Furthermore, according to the ethical permit received, we were able to access the complete health data of the patients but not of the controls. Thus, we were able to confirm that none of the OSCC patients were prescribed antibiotics or antifungals within 3 months prior to sample collection, but these or other medication data were not collected for the non-cancer controls, which is a limitation to the study, as some medications or medical conditions may affect the oral mycobiota either directly or through altering saliva secretion or mucosal stability.

The implication of this study is that the salivary mycobial profiles are statistically different when comparing OSCC patients with non-cancer controls. While a significantly larger dataset is needed to confirm the finding, our data suggest that the salivary fungal composition remains rather stable even amongst often radical environmental changes that occur during oral cancer treatment. This study also highlights the importance of determining fungal yield and how low fungal biomass may affect the validity of mycobiota results.

## Supplementary Material

Supplementary_Information.docx Supplementary_Information.docx

## Data Availability

The datasets supporting the conclusions of this article are available in the NCBI BioProject repository, PRJNA1494391 [https://www.ncbi.nlm.nih.gov/bioproject/1494391. Explanation on determination of *Candida* and *Malassezia* mycotypes is given in Supplementary information.
