# Supplementary material for: Salivary mycobial profiles of oral cancer patients – a cohort study
Source: J Oral Microbiol. 2026 Jul 30;18(1):2696598. doi: 10.1080/20002297.2026.2696598 (PMC13425524; doi:10.1080/20002297.2026.2696598)
Supplement: Supplementary_Information.docx [file ZJOM_A_2696598_SM0817.docx]

Supplementary information for *Salivary mycobial profiles of oral cancer patients*


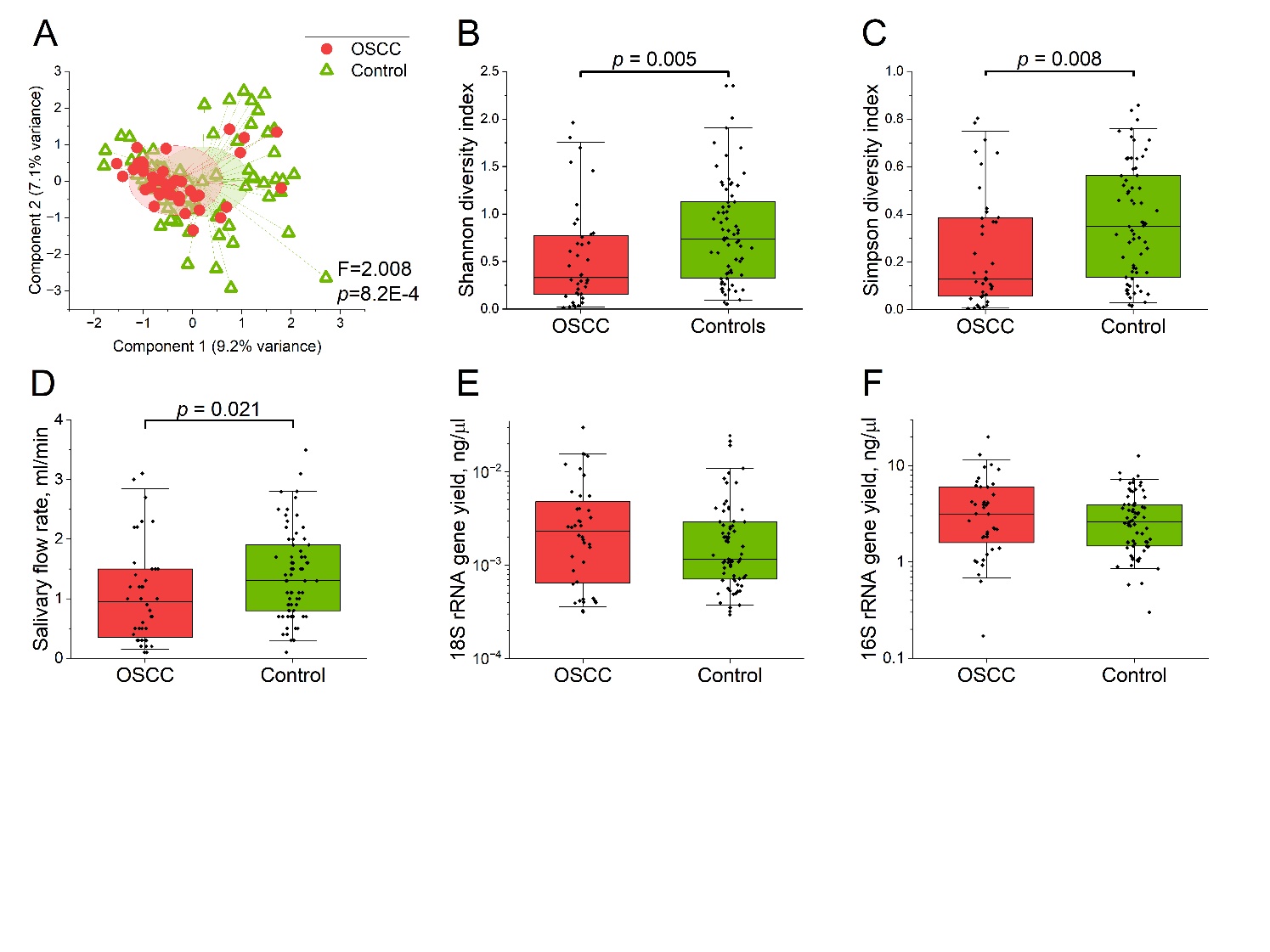
**Supplementary Figure 1** Comparisons between nonsmoker OSCC patients (n=40) and controls (n=70). **A** – Principal component analysis (PCA) plot of the pre-treatment patients and controls, F- and *p*-values from PERMANOVA. **B**, **C** – Median Shannon and Simpson’s diversity index values over 100 subsamples per sample; the difference is significant with both diversity indices (Independent-samples Mann-Whitney U). **D** – Salivary flow rate, which among controls is significantly higher (independent samples Mann-Whitney U). **E** – fungal DNA yield, no statistically significant difference between groups (independent samples Mann-Whitney U). **F** – Bacterial DNA yield, no statistically significant difference between groups (independent samples Mann-Whitney U).

**Supplementary Table 1** Association of confounders with two mycotypes within each group studied.

|  |  | *Candida* mycotype | *Malassezia* mycotype | Significance |
| --- | --- | --- | --- | --- |
| OSCC  (n=72) | Smokers | 30 | 3 | p = 0.510 |
|  | Non-smokers | 37 | 2 |  |
|  | Alcohol users^a^ | 43 | 3 | p = 0.890 |
|  | Alcohol non-users | 22 | 2 |  |
|  | Dentate | 58 | 5 | p = 0.381 |
|  | Edentate | 9 | 0 |  |
| Follow-up (n=25) | Smokers | 8 | 0 | p = 0.202 |
|  | Non-smokers | 9 | 2 |  |
|  | Alcohol users | 14 | 1 | p = 0.288 |
|  | Alcohol non-users | 3 | 1 |  |
|  | Dentate | 19 | 3 | p = 0.562 |
|  | Edentate | 3 | 1 |  |
| Control (n=78) | Smokers^b^ | 7 | 1 | p = 0.732 |
|  | Non-smokers | 53 | 14 |  |
|  | Alcohol users^c^ | 35 | 10 | p = 0.469 |
|  | Alcohol non-users | 26 | 5 |  |
|  | Dentate^d^ | 57 | 15 | p = 0.349 |
|  | Edentate | 4 | 0 |  |

^a^ = 2 OSCC patients did not report their alcohol use status (both had *Candida* mycotype); ^b^ = 3 controls did not report their smoking status (2 had *Candida* mycotype, 1 had *Malassezia* mycotype); ^c^ = 2 controls did not report their alcohol use status (one of each mycotype); ^d^ = 2 controls did not report whether they were dentate or edentate (one of each mycotype).

**Supplementary Note 1: Determination of the *Candida* and *Malassezia* mycotypes**

In determining the two mycotypes, first the OTUs assigned as either genus *Candida* or genus *Malassezia* according to the UNITE9 database were included, which amounted to 21 OTUs assigned genus *Candida* and 19 OTUs assigned genus *Malassezia*. Additionally, 10 clinically relevant species, that were previously known as belonging to genus *Candida*, were included as determinants of the *Candida* mycotype (Supplementary Table 2).

**Supplementary Table 2** Current and previous names of clinically relevant species included as determinants of the *Candida* mycotype.

| **OTU** | **Current species name** | **Previous species name** | **Reference** |
| --- | --- | --- | --- |
| 7 | *Nakaseomyces glabrata* | *Candida glabrata* | 1 |
| 8 | *Debaryomyces hansenii* | *Candida famata* | 1 |
| 9 | *Pichia kudriavzevii* | *Candida krusei* | 1 |
| 28 | *Meyerozyma guilliermondii* | *Candida guilliermondii* | 1 |
| 42 | *Kluyveromyces marxianus* | *Candida kefyr, Candida pseudotropicalis* | 1 |
| 101 | *Wickerhamomyces anomalus* | *Candida pelliculosa, Pichia anomala* | 1 |
| 118 | *Diutina neorugosa* | *Candida neorugosa* | 1 |
| 142 | *Clavispora lusitaniae* | *Candida lusitaniae* | 1 |
| 319 | *Wickerhamiella pararugosa* | *Candida pararugosa* | 2 |
| 472 | *Nakaseomyces glabrata* | *Candida glabrata* | 1 |

Additionally, after performing NCBI BLAST on the OTUs that did not have a genus or species-level assignment, OTU11 *Candida sake* and OTU21 *Candida zeylanoides* were added as part of the Candida genus. All in all, 33 OTUS were determined to represent genus *Candida* and 19 OTUs belonged to genus *Malassezia*.

After determining which OTUs represented either genus *Candida* or genus *Malassezia*, the total relative abundancies of each genus were summed. When the total relative abundance of genus *Candida* in a sample was more than 5% larger than the total relative abundance of genus *Malassezia*, the sample was assigned the *Candida* mycotype. The samples which had a total relative abundance of genus *Malassezia* larger than 5% compared with the total relative abundance of genus *Candida* were assigned the *Malassezia* mycotype.

Supplementary references

1. Kidd SE, Abdolrasouli A, Hagen F. Fungal Nomenclature: Managing Change is the Name of the Game. Open Forum Infectious Diseases. 2023;10(1). doi:10.1093/ofid/ofac559

2. Murata S, Mimura K, Kawamura T, Saito H, Ohno H, Tsujii E, Shinohara T, Miyazaki Y, Ohki T. Bloodstream infection caused by Wickerhamiella pararugosa in a patient with intestinal obstruction: A case report. Journal of Infection and Chemotherapy. 2024 Feb 16. doi:10.1016/J.JIAC.2024.02.014

**Supplementary Note 2:** **STROBE Statement—checklist of items that should be included in reports of observational studies**

|  | Item No | Recommendation | Line No |
| --- | --- | --- | --- |
| **Title and abstract** | 1 | (*a*) Indicate the study’s design with a commonly used term in the title or the abstract |  |
|  |  | (*b*) Provide in the abstract an informative and balanced summary of what was done and what was found | **35-45** |
| Introduction | | |  |
| Background/rationale | 2 | Explain the scientific background and rationale for the investigation being reported | **56-63** |
| Objectives | 3 | State specific objectives, including any prespecified hypotheses | **64-71** |
| Methods | | |  |
| Study design | 4 | Present key elements of study design early in the paper | **64-69,**  **Figure 1** |
| Setting | 5 | Describe the setting, locations, and relevant dates, including periods of recruitment, exposure, follow-up, and data collection | **208-222** |
| Participants | 6 | (*a*) *Cohort study*—Give the eligibility criteria, and the sources and methods of selection of participants. Describe methods of follow-up  *Case-control study*—Give the eligibility criteria, and the sources and methods of case ascertainment and control selection. Give the rationale for the choice of cases and controls  *Cross-sectional study*—Give the eligibility criteria, and the sources and methods of selection of participants | **210-212, 214-215, 220-222** |
|  |  | (*b*) *Cohort study*—For matched studies, give matching criteria and number of exposed and unexposed  *Case-control study*—For matched studies, give matching criteria and the number of controls per case |  |
| Variables | 7 | Clearly define all outcomes, exposures, predictors, potential confounders, and effect modifiers. Give diagnostic criteria, if applicable | **278-287** |
| Data sources/ measurement | 8* | For each variable of interest, give sources of data and details of methods of assessment (measurement). Describe comparability of assessment methods if there is more than one group | **223-226, 228-229** |
| Bias | 9 | Describe any efforts to address potential sources of bias |  |
| Study size | 10 | Explain how the study size was arrived at | **209, 214-218, 235, 271-276** |
| Quantitative variables | 11 | Explain how quantitative variables were handled in the analyses. If applicable, describe which groupings were chosen and why | **278-302** |
| Statistical methods | 12 | (*a*) Describe all statistical methods, including those used to control for confounding | **278-295** |
|  |  | (*b*) Describe any methods used to examine subgroups and interactions | **95-104** |
|  |  | (*c*) Explain how missing data were addressed | **274-276, 282** |
|  |  | (*d*) *Cohort study*—If applicable, explain how loss to follow-up was addressed  *Case-control study*—If applicable, explain how matching of cases and controls was addressed  *Cross-sectional study*—If applicable, describe analytical methods taking account of sampling strategy | **274-276**  **220-222** |
|  |  | (*e*) Describe any sensitivity analyses | **Not included** |

Continued on next page

| Results | | | Line No |
| --- | --- | --- | --- |
| Participants | 13* | (a) Report numbers of individuals at each stage of study—eg numbers potentially eligible, examined for eligibility, confirmed eligible, included in the study, completing follow-up, and analysed | **Figure 1** |
|  |  | (b) Give reasons for non-participation at each stage | **215-217, 235, 271-276** |
|  |  | (c) Consider use of a flow diagram | **Figure 1** |
| Descriptive data | 14* | (a) Give characteristics of study participants (eg demographic, clinical, social) and information on exposures and potential confounders | **Table 1** |
|  |  | (b) Indicate number of participants with missing data for each variable of interest | **Table 1** |
|  |  | (c) *Cohort study*—Summarise follow-up time (eg, average and total amount) | **215** |
| Outcome data | 15* | *Cohort study*—Report numbers of outcome events or summary measures over time | **Table 1** |
|  |  | *Case-control study—*Report numbers in each exposure category, or summary measures of exposure | **Table 1** |
|  |  | *Cross-sectional study—*Report numbers of outcome events or summary measures | **N/A** |
| Main results | 16 | (*a*) Give unadjusted estimates and, if applicable, confounder-adjusted estimates and their precision (eg, 95% confidence interval). Make clear which confounders were adjusted for and why they were included | **Figures 2-4** |
|  |  | (*b*) Report category boundaries when continuous variables were categorized | **N/A** |
|  |  | (*c*) If relevant, consider translating estimates of relative risk into absolute risk for a meaningful time period | **N/A** |
| Other analyses | 17 | Report other analyses done—eg analyses of subgroups and interactions, and sensitivity analyses | **Supplementary information** |
| Discussion | | |  |
| Key results | 18 | Summarise key results with reference to study objectives | **145-156, 161, 168-169** |
| Limitations | 19 | Discuss limitations of the study, taking into account sources of potential bias or imprecision. Discuss both direction and magnitude of any potential bias | **201-202** |
| Interpretation | 20 | Give a cautious overall interpretation of results considering objectives, limitations, multiplicity of analyses, results from similar studies, and other relevant evidence | **Discussion** |
| Generalisability | 21 | Discuss the generalisability (external validity) of the study results | **162-163, 172-174** |
| Other information | | |  |
| Funding | 22 | Give the source of funding and the role of the funders for the present study and, if applicable, for the original study on which the present article is based | **279-283** |

*Give information separately for cases and controls in case-control studies and, if applicable, for exposed and unexposed groups in cohort and cross-sectional studies.

**Note:** An Explanation and Elaboration article discusses each checklist item and gives methodological background and published examples of transparent reporting. The STROBE checklist is best used in conjunction with this article (freely available on the Web sites of PLoS Medicine at <http://www.plosmedicine.org/>, Annals of Internal Medicine at <http://www.annals.org/>, and Epidemiology at <http://www.epidem.com/>). Information on the STROBE Initiative is available at www.strobe-statement.org.
